# Influence of a University Tobacco-Free Policy on the Attitudes, Perceptions of Compliance, and Policy Benefit Among the University Students: A Pre-Post Investigation

**DOI:** 10.3389/ijph.2021.614602

**Published:** 2021-04-29

**Authors:** Monique Chaaya, Dina Farran, Dahlia Saab, Mahmoud Al-Hindi, Maya Romani, Mary Khairallah, Rima Nakkash

**Affiliations:** ^1^ Faculty of Health Sciences, American University of Beirut, Beirut, Lebanon; ^2^ Faculty of Medicine, American University of Beirut, Beirut, Lebanon; ^3^ Department of Mechanical Engineering, Maroun Semaan Faculty of Engineering and Architecture, American University of Beirut, Beirut, Lebanon; ^4^ Department of Family Medicine, American University of Beirut Medical Center, Beirut, Lebanon; ^5^ Human Resources Department, American University of Beirut, Beirut, Lebanon

**Keywords:** tobacco-free policy, university, students, attitude, smoking behavior

## Abstract

**Objective:** To evaluate the effectiveness of a university tobacco-free policy by examining differences in students’ attitudes, perceptions of compliance and policy benefits, after one year of the policy’s implementation.

**Methods:** Cross-sectional studies were undertaken to collect data pre- and 1 year post-policy implementation. The two samples were selected using stratified random sampling.

**Results:** The prevalence of smoking decreased from 26% pre-policy implementation to 21% 1 year after (*p* = 0.035). The proportion of smokers who thought the policy had contributed to a reduction in smoking frequency increased from 10% to 70% (*p* < 0.001). Smokers’ support for the policy rose from 42 to 58% (*p* = 0.007).

**Conclusion:** Against the background of a strongly pro-tobacco environment in Lebanon, it is possible to create a positive change in the mindset of smokers at the levels of the education and smoking cessation and more efforts should be expended to bring it about.

## Introduction

Tobacco use is a major contributor to a multitude of diseases, including atherosclerotic cardiovascular disease, lung cancer, asthma and chronic obstructive pulmonary disease (COPD) [1]. These tobacco-related illnesses are not only associated with daily tobacco use or a lifetime of smoking, but also affect people exposed to secondhand smoke (SHS) who are as susceptible to the health risks of tobacco dependence as smokers [2]. Although tobacco use is declining worldwide, it increased in Africa and the Eastern Mediterranean Region over the last decade [3].

Although Lebanon ratified the WHO Framework Convention on Tobacco Control in 2005, the government did not adopt a tobacco control policy until 2011. Law 174 bans smoking in public places, on public transport, at workplaces and outdoors at schools, universities, and health and sports facilities. However, the law was enforced inconsistently, or not at all, reflecting the absence of political will for tobacco control and opposition from allies of the tobacco industry in the government [4]. In 2015, the WHO estimated cigarette smoking prevalence in Lebanon stood at 32% among males aged 18 and over, and 21% among females aged 18 and over [5].

In a cross-sectional survey assessing students’ attitudes towards a tobacco ban at a private university in Lebanon (AUB), Chaaya et al. reported that 25% of participants started smoking after joining the university [6]. Smoking prevalence among university students in the Middle East is relatively high with males showing higher rates than females. A cross-sectional study carried out at Aleppo University in Syria showed that 31% of male and 7% of female students were regular smokers [7]. Similarly, a pilot study at University of Jordan reported that smoking prevalence among college students was 27% [8]. In Saudi Arabia, a systematic review and meta-analysis of tobacco prevalence among college students estimated the rate to be 17% (26% in males and 5% in females) [9].

Recently, there has been a surge in the adoption of tobacco control policies at universities. Evaluations of these policies show a correlation between how comprehensive the policy is and its success in decreasing smoking behavior [10–12]. A complete ban of tobacco use in universities was shown to be associated with lower self-reported intentions to smoke and a reduction in pro-smoking attitudes, smoking frequency, and exposures to second hand smoke [10–14]. Seo et al. reported that, 1 year post-implementation of a smoke-free policy at a college in Indiana, undergraduates’ smoking prevalence was significantly lower than before the policy (16.5%–12.8%) [12]. They also noted that the perception of peers’ compliance increased after implementation. Another cross-sectional study, evaluating students’ attitudes, beliefs and smoking behavior over 4 years following a tobacco ban, showed that smoking prevalence decreased from 9.5% before implementation to 7% 3 years after the ban [11]. Lupton and Townsend found in their systematic review and meta-analysis that 59% of college students supported smoke-free policies and that the support increased with policy implementation [15].

**FIGURE 1:**
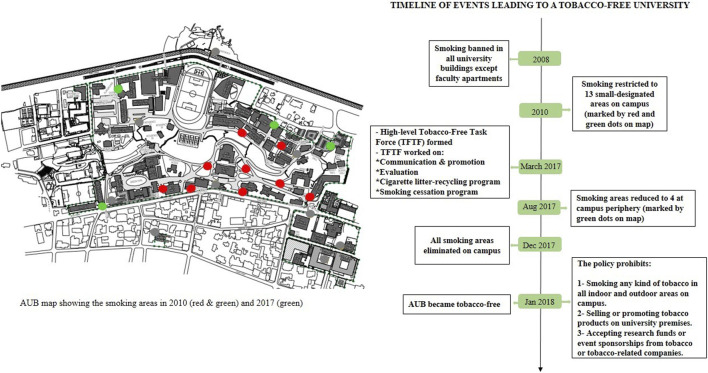
Timeline of events leading to a tobacco-free university (Lebanon, 2008-2018)

**FIGURE 2:**
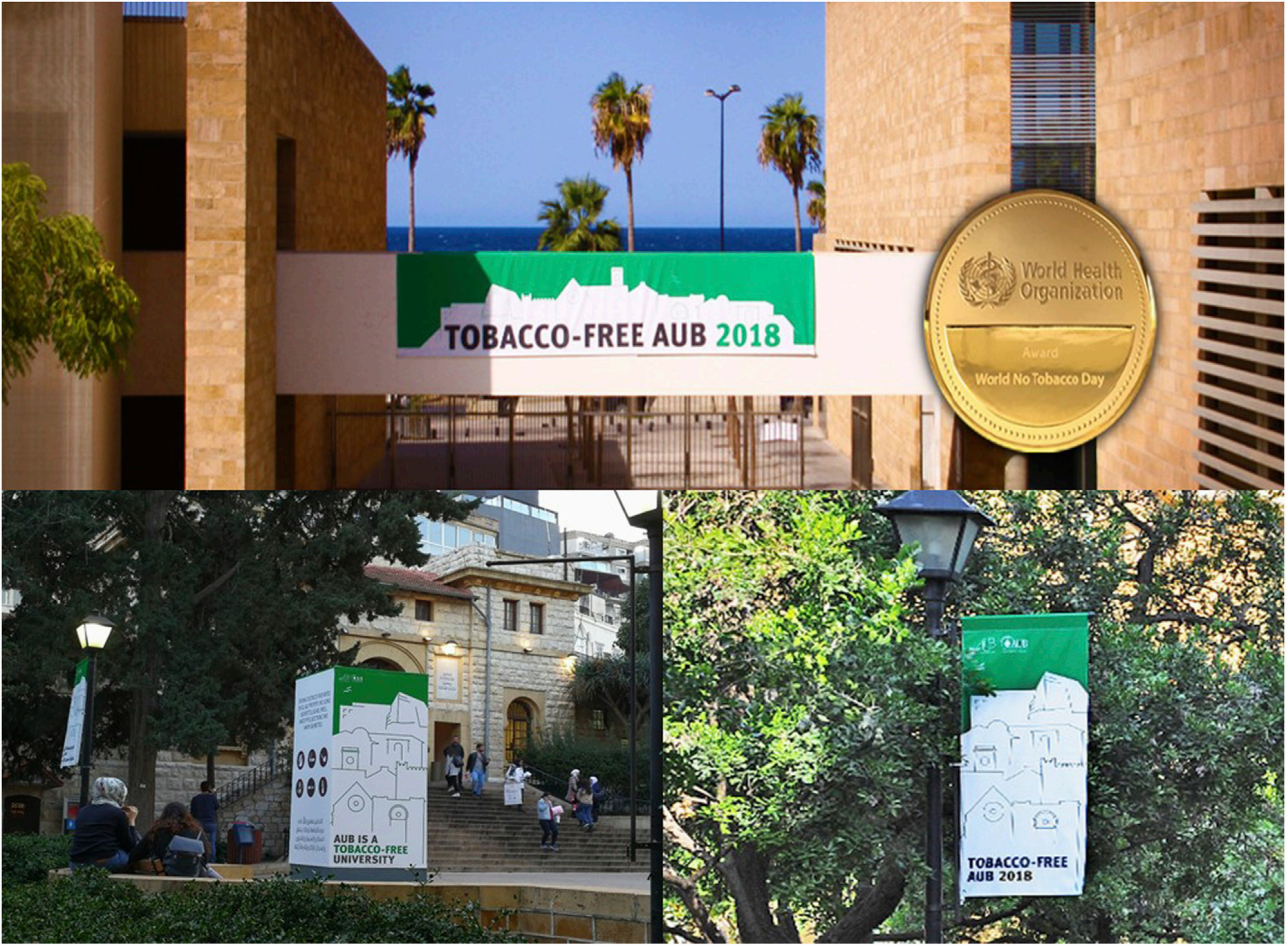
Communication of the tobacco-free policy.

The number of universities in the Middle East going tobacco-free is lower than elsewhere around the world, and no published studies have yet evaluated their effectiveness in the region [16]. The American University of Beirut (AUB), one of the largest universities in Lebanon, started its tobacco-free policy initiative in the year 2000 by banning smoking in all university buildings except faculty and staff apartments. In 2008, smoking was restricted to a number of confined designated areas. In March 2017, a high-level task force was assigned by the president of the university to work toward the adoption of a tobacco-free policy and plan for implementation, enforcement, and evaluation [17]. The task force developed an initial plan to reduce the number of designated areas from 13 throughout the campus to 4 at the peripheries, before the removal of all designated areas several months later. AUB implemented its tobacco-free policy in January 2018, and succeeded in enforcing it despite the contextual challenges. It was the first Lebanese university to implement Law 174 banning smoking in all educational institutions (Figures 1, 2).

In this manuscript, we report on the evaluation of the AUB tobacco-free policy by comparing independent samples of students pre- and 1 year post-policy implementation. Specifically, the study examined: 1) changes in students’ attitudes, perceptions of policy benefits and perceptions of compliance among smokers and non-smokers 1 year post-policy implementation; 2) changes in smokers’ attitudes, perceptions of policy benefits and perceptions of compliance 1 year post-policy implementation; 3) changes in smokers’ behavior one year post-policy implementation. The evaluation of AUB tobacco-free policy was funded by the university presidents’ initiative.

## Methods

The Institutional Review Board at AUB approved the research protocol. Participants were guaranteed anonymity.

### Participants

Two cross-sectional surveys were conducted: the first prior to implementation of the tobacco-free policy (November 2017) and the second 1 year post-policy implementation (November 2018). The process of data collection among students took around two weeks in both years. The targeted sample size at each data collection time was 2,172 students, which approximately equaled 23% of the total number of students in each year (9,143 and 9,404 in AY17/18 and 18/19 respectively). A list of the core courses attended by the students in the fall term of each academic year was obtained from the Office of Student Affairs. A sample of graduate and undergraduate courses was randomly selected by the statistician at the Office of the Registrar based on stratified cluster sampling, with faculties being the stratification variable to ensure representation of all students. Professors of the selected courses were contacted by email to seek their approval for data collection from students during class time. The main reason for refusals in both periods was the timing of the data collection which coincided with the last few sessions of the term. The courses for which data collection was approved were visited by two research team members and all students present were asked to complete the survey. The sample sizes reached pre- and 1 year post-implementation of the policy were 809 and 615 students respectively, with an overall response rate of 37 and 28%. The distribution of the final sample in both surveys was comparable to the AUB student population in terms of gender, level, and Faculty (Data not shown).

### Survey and Data Collection

A self-administered paper-and-pencil survey was designed to collect data within 5–7 min. Participation was voluntary and no identifiable data were collected. The survey instrument collected data on demographics (gender, age, marital status, class, faculty, and place of residence), students’ attitudes toward the tobacco-free policy, their perceptions of compliance, their perceptions of the policy benefits, and their smoking behavior. Students were asked to indicate the extent to which they supported the policy, whether they thought policy should have exceptions, whether it created a healthy environment, promoted quit attempts had an affect on the neighborhood. Responses were reported on a 3-point Likert scale (“large extent,” “some extent” and “not at all,” with a “not sure” option). Students were also asked to what extent they believed that the policy resulted in a reduction in students’ smoking frequency and a reduction in the rate of student absences. Responses were reported on a 4-point Likert scale (“not a benefit,” “minor benefit,” “moderate benefit” and “major benefit,” with a “don’t know” option). In addition, questions on smoking status, smoking history, quit attempts and change in smoking behavior following implementation of the tobacco-free policy were included in the survey instrument.

### Data Analysis

Univariate analyses were done to describe basic demographic and smoking characteristics. To examine differences in attitudes, compliance and smoking behavior of students pre- and post-policy implementation, bivariate analyses by year and smoking status were performed. χ^2^ tests were computed to determine the statistically significant differences, identified at a *p* value < 0.05. Smoking status, initially grouped into four categories (never smoked, daily, former and occasional smoker) was dichotomized into smokers (daily and occasional smokers) and non-smokers (never smoked and former smoker). The Likert scale responses for the questions related to attitudes, compliance, and perceived benefits, were collapsed to two categories. For responses to the attitude questions, “large extent” and “some extent” were considered as “Yes,” while “not at all” and “not sure” were considered as “No.” For perception of compliance and benefits: “minor benefit,” “moderate benefit” and “major benefit” were considered as “Yes,” while “not a benefit” and “don’t know” were considered as “No.” The analysis aimed, first, to compare attitudes, compliance and perceived benefits among smokers and non-smokers pre- and 1 year post-policy implementation and, second, to study the change in smokers’ attitudes, compliance, perceived benefits and smoking behavior 1 year after policy implementation. Samples pre- and 1 year post-policy implementation were independent.

## Results

Overall, 809 completed the questionnaire in the first cross-sectional study (pre-tobacco-free policy implementation) and 615 in the second study (post-tobacco-free policy implementation). The mean age of students was 20 years in both samples; the proportion of females was 56% in the first survey and 55% in the second. (Table 1). The distribution of students by faculties was similar in both years (data not shown).

**TABLE 1:** Students’ characteristics pre- and 1-year post-policy implementation.

	Pre-implementation	Post-implementation	*p* value
N	809	615	
Female	451 (56.0)	340 (55.4)	0.849
Age [mean(SD)]	20.38 (3.13)	20.68 (2.90)	0.069
Smoking status			
Never	550 (70.7)	469 (76.3)	
Daily	94 (12.1)	62 (10.1)	
Former	26 (3.3)	17 (2.8)	
Occasional	108 (13.9)	67 (10.9)	
Smokers (daily and occasional)	202 (26.0)	129 (21.0)	0.035

The majority of students reported that they had never smoked (71 pre- and 76% 1-year post-policy implementation). The proportion of former smokers was the smallest category in both years, not exceeding 4%. Almost 14% of students identified themselves as occasional smokers before policy implementation compared to 11% 1 year after (Table 1). After combining the “daily” and “occasional” categories, the proportion of smokers showed a statistically significant decrease from 26% pre-to 21% (*p* = 0.035) 1-year post-policy implementation.

### Attitude Toward the Tobacco-Free Policy

Smokers and non-smokers’ attitudes toward the tobacco-free policy were compared pre- and 1 year post-implementation (Table 2). Overall, the difference in attitude was statistically significant with non-smokers showing more positivity and support at both time points (Table 2).

**TABLE 2:** Nonsmokers’ and Smokers’ attitude and perception of policy benefits pre- and 1 year post-implementation.

	Pre-implementation			Post-implementation		*p* value
	Non-smokers	Smokers	*p* value	Non-smokers	Smokers	
n	576	202		486	129	
Attitude toward the policy						
Support the policy	485 (84.2)	85 (42.3)	<0.001	440 (90.5)	75 (58.1)	<0.001
Policy has created a healthy environment	502 (87.8)	127 (63.2)	<0.001	408 (84.0)	79 (61.2)	<0.001
There should be exceptions	238 (46.6)	66 (35.7)	0.013	111 (22.8)	85 (65.9)	<0.001
The policy had a negative effect on neighborhood^a^	151 (37.9)	68 (58.6)	<0.001	212 (60.1)	75 (83.3)	<0.001
Perception of policy benefits						
Policy has reduced smoking frequency	52 (9.1)	20 (10.0)	0.811	395 (81.3)	90 (69.8)	0.006
Policy has decreased the rate of student absences	79 (13.8)	26 (13.1)	0.892	236 (48.6)	34 (26.4)	<0.001
Perception of compliance with the policy						
Students are compliant	439 (77.7)	132 (67.7)	0.007	390 (80.2)	104 (80.6)	0.999
Staff are compliant	446 (78.1)	141 (70.5)	0.038	356 (73.3)	91 (70.5)	0.615
Faculty members are compliant	467 (81.5)	133 (66.8)	<0.001	362 (74.5)	89 (69.0)	0.253

^a^ Numbers of Non-smokers (NS) and Smokers (S) reporting that the policy had an effect on the neighborhood: N applicable for 2017: NS = 403/S = 117; for 2018: NS = 405/S = 38.

Smokers’ attitudes before and after policy implementation were compared. It was notable that the proportion of smokers supporting the policy statistically significantly increased by 16%, from 42 pre-to 58% at 1-year post-implementation (*p* = 0.007). However, a higher proportion of smokers believed that the policy had negatively affected the neighborhood and the difference was statistically significant (< 0.001) (Table 3). The negative effect that the policy had on the neighborhood was perceived as littering and crowding at university entrances. Students expressed these concerns in informal discussions as well as in the university student newsletter. Among non-smokers, more support was expressed at 1-year follow up (*p* = 0.003) and a significantly lower proportion believed that the policy should have exceptions (*p* < 0.001).

**TABLE 3:** Smokers’ and non-smokers’ change in attitude and perception of policy benefits.

	Non-smokers			Smokers		
Pre-	1-year post-	*p* value	Pre-	1-year post-	*p* value
n	576	486		202	129	
Attitude toward the policy						
Support the policy	485 (84.2)	440 (90.5)	0.003	85 (42.3)	75 (58.1)	0.007
Policy has created a healthy environment	502 (87.8)	408 (84.0)	0.091	127 (63.2)	79 (61.2)	0.811
Policy has promoted quit attempts	388 (67.8)	255 (52.5)	<0.001	89 (44.3)	40 (31.0)	0.022
There should be exceptions	238 (46.6)	111 (22.8)	<0.001	143 (71.1)	85 (65.9)	0.376
The policy had a negative effect on neighborhood^a^	151 (37.9)	212 (60.1)	<0.001	68 (58.6)	75 (83.3)	<0.001
Perception of policy benefits						
Policy has reduced smoking frequency	52 (9.1)	395 (81.3)	<0.001	20 (10.0)	90 (69.8)	<0.001
Policy has decreased the rate of student absences	79 (13.8)	236 (48.6)	<0.001	26 (13.1)	34 (26.4)	0.004
Perception of compliance with the policy						
Students are compliant	439 (77.7)	390 (80.2)	0.351	132 (67.7)	104 (80.6)	0.015
Staff are compliant	446 (78.1)	356 (73.3)	0.077	141 (70.5)	91 (70.5)	0.999
Faculty members are compliant	467 (81.5)	362 (74.5)	0.007	133 (66.8)	89 (69.0)	0.774

^a^ Numbers of Non-smokers (NS) and Smokers (S) reporting that the policy had an effect on the neighborhood: for non-smokers N applicable 2017 = 403/N applicable 2018 = 353; for smokers: N applicable 2017 = 54/N applicable 2018 = 38.

### Perceived Benefits of the Tobacco-Free Policy

The difference in reported perceived benefits between non-smokers and smokers 1 year after implementation of the policy was statistically significant, with non-smokers having favorable perceptions of the benefits of the policy in relation to a reduction in smoking frequency and a decreased rate of students absences (*p* = 0.006 and *p* < 0.001 respectively) (Table 2).

A major difference in smokers’ perception of the benefits of the policy was noted when comparing pre- and post-policy implementation. The proportion of smokers who thought the policy had contributed to a reduction in smoking frequency increased seven times in 1 year, from 10% before implementation rising to 70% 1-year after (*p* < 0.001). In addition, 26% of smokers in 2018 reported that the policy had been beneficial in decreasing their rate of absences compared to 13% the previous year (*p* = 0.004). (Table 3).

### Compliance and Smoking Behavior

Students’ perception of compliance was also assessed 1-year post-policy implementation. There was no significant difference between smokers and non-smokers. The majority of students reported compliance by their peers, staff and faculty (Table 2). Similar results were obtained when comparing smokers’ perceptions of compliance pre- and 1-year post-implementation, except that the proportion of students perceiving their peers as compliant with the policy increased statistically significantly from 68 to 81% (*p* = 0.015) (Table 3).

As for smoking behavior, the proportion of daily smokers who had started smoking after joining AUB changed from 27% pre-implementation to 21% 1-year later. The proportion of smokers thinking of quitting in the next six months changed from 58 to 69%. However, the differences were not statistically significant. Around 8% of smokers joined the smoking cessation program offered by the Wellness Program at the AUB Medical Center and 11% of smokers reported a decrease in their off-campus (i.e., home) smoking behavior following policy implementation (Table 4).

**TABLE 4:** Students’ smoking behavior pre- and 1-year post-policy implementation.

	Pre- implementation	Post-implementation	*p* value
n	94	62	
Started smoking after joining	25 (27.2)	13 (21.3)	0.528
Thinking of quitting in the next 6 months	53 (58.2)	43 (69.4)	0.22
Consider joining the smoking cessation program	35 (37.6)	25 (40.3)	0.866
Joined the smoking cessation program	NA	5 (8.1)	—
Decreased their off-campus smoking behavior	NA	7 (11.3)	—

Note: Percentages may not precisely reflect the figures as there were few missing values. Percentages in this table represent regular smokers only (excluding the occasional smokers).

## Discussion

This study is among the first to evaluate the effectiveness of a university tobacco-free policy in the Eastern Mediterranean Region, where national tobacco control laws are only weakly enforced or implemented. The prevalence of daily smoking (12% pre-implementation and 10% 1-year later) was lower than reported in other cross-sectional studies on tobacco use in private Lebanese universities (18–19%), although it was similar to a study conducted at AUB in 2008 [6, 18, 19].

The proportion of occasional smokers in both surveys was higher than that of daily smokers, which is a concern, given the evidence that occasional smoking can lead to daily smoking [20]. As for the decrease in smoking prevalence 1-year post policy implementation (26–21%), it was consistent with other studies evaluating changes in smoking prevalence following complete tobacco bans [11, 12]. In college campuses partially banning tobacco, it was found that permitting smoking in designated areas was associated with a higher rate of smoking, owing to the fact that the interaction in such places increased the perceived rewards of being a smoker and led to an increased frequency of visits [10, 21, 22].

The proportion of smokers supporting the tobacco-free policy significantly increased 1 year after the implementation, while non-smokers showed a favorable attitude toward the policy in both years. These results are in-line with other studies showing that a majority of students, including smokers, support tobacco control policies and they tend to show more support after policy implementation [11, 12, 23]. Smokers, however, still prefer to have designated smoking areas on campus, while non-smokers think there should be no exceptions to the policy. This difference is attributable to the fact that smokers sometimes question the value of banning tobacco in outdoor areas, believing that they have a right to smoke in open spaces and it is not harmful for others [24]. On the other hand, the proportion of smokers believing the policy reduced the frequency smoking significantly increased at 1 year follow up, which is in line with many previous studies [10–12].

Smoking behavior was assessed after 1 year of the implementation of the policy through changes in off-campus smoking behavior and intentions to quit or join smoking cessation programs. In this study, occasional smokers were not asked about quitting behavior, as it was previously reported that most occasional smokers do not consider themselves addicted to tobacco and are less likely to report quitting or joining a cessation program [28]. Results showed that fewer students started smoking after joining AUB in 2018 and that a higher proportion of smokers are thinking of quitting within the next 6 months compared to the pre-policy implementation. However, the differences were not statistically significant, which may be attributed to the social norms associated with smoking in Lebanon or to the fact that a year is a relatively short period to change a behavior. In addition, one in ten smokers reported a decrease in their off-campus smoking behavior, which is consistent with a study showing that legislative smoking bans in public places influence social norms related to smoking and thus encourage people to adopt similar restrictions at home [25]. A small number of smokers joined the smoking cessation program and data on whether their quit attempts were successful is not available. Previous studies reported that policies coupled with smoking cessation programs led to increased treatment-seeking behavior and quit attempts [26, 27]. A study done by Hahn et al. found that 16 months after the implementation of the policy, 9% of the smokers who used the smoking cessation program reported they had quit smoking [26]. However, according to Moran, students considering themselves social or occasional smokers were less likely to participate in such programs and quit, as they did not consider themselves addicted to cigarette smoking [28].

This study has some limitations that deserve discussion. Firstly, its cross-sectional nature precludes any causal association between the tobacco-free policy and smoking behavior. Secondly, it relies on self-reporting, which may introduce response bias, while using a self-administered questionnaire with clearly constructed questions would minimize information bias and ensure confidentiality and privacy. Thirdly, it covers only one university, so the findings may not be generalizable to the entire Lebanese college student community. Fourthly, selection bias due to non-response is a major concern in cross-sectional studies. Because many professors did not allow us to do data collection during their class time, we were not able to reach the sample size originally targeted. Still, in our study, the response rate is comparable to that reported in similar studies [27, 29, 30], and the distribution of those who responded was not significantly different from the original population, as noted in the methods section. Finally, we would like to note that in our context occasional smokers don’t consider themselves as smokers (we defined occasional smokers as those smoking less than one cigarette per day), thus we did not opt to ask them about their quit attempts and intentions to quit. However, exclusion of occasional smokers may be seen as insufficient in providing evidence on the decrease of smoking initiation after joining AUB.

The study also has some noteworthy strength. It is the first in the Middle East to assess changes in students’ attitudes, perceptions and smoking behavior following the implementation of a tobacco-free policy. The large sample size in both rounds gave the study sufficient power to detect changes after implementation of the policy. In addition, the sampling frame included students from all faculties and levels, ensuring representativeness. Finally, this study could set the stage for similar research in the region.

The AUB tobacco-free policy represents a remarkable form of compliance and enforcement of national tobacco control legislation. This study assesses changes in students’ attitudes, perceptions and smoking behavior 1 year after the transition of their university campus from partially to wholly tobacco-free. Overall, attitudes toward the policy and perception of its benefits improved among smokers 1 year after implementation. As for smoking behavior, a drop in smoking rate was noted.

Knowing that smoking prevalence is increasing among college students, great efforts should be expended at both the educational level and on cessation programs to ensure a positive change in smokers’ mindsets. College years are critical to protect this age group, to educate young adults about the adverse effects of smoking and more importantly to provide them with supportive, smoke-free environments and access to cessation support when needed. As other Lebanese and Arab universities adopt tobacco-free policies, more research should be done in this field to develop generalizable conclusions about the effectiveness of tobacco control policies in countries known for their pro-smoking environment.

## Data Availability

The raw data supporting the conclusions of this article will be made available by the authors, without undue reservation.
